# Supplementary material for: An open-source framework for large-scale, flexible evaluation of biomedical text mining systems
Source: J Biomed Discov Collab. 2008 Jan 29;3:1. doi: 10.1186/1747-5333-3-1 (PMC2276192; doi:10.1186/1747-5333-3-1)
Supplement: Additional file 1 — Evaluation platform source code. The additional file contains the software evaluation framework discussed in this paper. The most current release can be downloaded from SourceForge.net [1]. See the accompanying README file for instructions on installation and use. Also included in the distribution is a collection of UIMA wrappers for some commonly used BioNLP tools and annotated corpora. [file 1747-5333-3-1-S1.zip › bionlp_uima_repository-1.0.1/docs/api/src-html/edu/uchsc/ccp/uima/ae/nlp/anaphora/ProteinAnaphoraResolver.html]

```
001    package edu.uchsc.ccp.uima.ae.nlp.anaphora;
002    
003    import java.util.ArrayList;
004    import java.util.Iterator;
005    import java.util.List;
006    
007    import org.apache.uima.analysis_engine.ResultSpecification;
008    import org.apache.uima.analysis_engine.annotator.AnnotatorProcessException;
009    import org.apache.uima.analysis_engine.annotator.JTextAnnotator_ImplBase;
010    import org.apache.uima.cas.CASException;
011    import org.apache.uima.jcas.JCas;
012    
013    import edu.uchsc.ccp.uima.annotation.CCPDocumentInformation;
014    import edu.uchsc.ccp.uima.annotation.CCPSemanticAnnotation;
015    import edu.uchsc.ccp.uima.annotation.CCPTextAnnotation;
016    import edu.uchsc.ccp.uima.util.UIMA_Util;
017    import edu.uchsc.ccp.util.nlp.annotation.InvalidSpanException;
018    import edu.uchsc.ccp.util.nlp.annotation.Span;
019    import edu.uchsc.ccp.util.nlp.mention.ClassMentionTypes;
020    
021    /**
022     * This class is based on a simple anaphora algorithm that Z came up with.
023     * 
024     * @author williamb
025     * 
026     */
027    public class ProteinAnaphoraResolver extends JTextAnnotator_ImplBase {
028    
029        private final boolean DEBUG = false;
030        
031        private List<String> singularPronounList;
032    
033        private List<String> pluralPronounList;
034    
035        private final int ADD_FIRST_SINGULAR = 0;
036    
037        private final int ADD_LAST_SINGULAR = 1;
038    
039        private final int ADD_FIRST_PLURAL = 2;
040    
041        private final int ADD_LAST_PLURAL = 3;
042        
043        private List<CCPTextAnnotation> proteinsToAddToCAS;
044    
045        public void process(JCas jcas, ResultSpecification arg1) throws AnnotatorProcessException {
046    
047            proteinsToAddToCAS = new ArrayList<CCPTextAnnotation>();
048            
049            singularPronounList = new ArrayList<String>();
050            pluralPronounList = new ArrayList<String>();
051            singularPronounList.add("it");
052            singularPronounList.add("its");
053            pluralPronounList.add("they");
054            pluralPronounList.add("their");
055    
056            /* Iterate over all tokens looking for pronouns */
057            Iterator annotationIter = jcas.getJFSIndexRepository().getAnnotationIndex(CCPTextAnnotation.type).iterator();
058            while (annotationIter.hasNext()) {
059                CCPTextAnnotation annot = (CCPTextAnnotation) annotationIter.next();
060                if (annot.getClassMention().getMentionName().equals(ClassMentionTypes.TOKEN)) {
061                    /* If the annotation is a token */
062                    String tokenText = annot.getCoveredText();
063                    if (singularPronounList.contains(tokenText.toLowerCase()) || pluralPronounList.contains((tokenText.toLowerCase()))) {
064                        /* If the token is a pronoun in the pronounList, then attempt to assign a previous protein reference to the pronoun */
065                        if (DEBUG) {
066                            System.out.println("Found pronoun token: \"" + tokenText + "\"");
067                        }
068                        addUpstreamProteinReferences(annot, jcas);
069            
070                    }
071                }
072            }
073            
074            for (CCPTextAnnotation ccpTA : proteinsToAddToCAS) {
075                    ccpTA.addToIndexes();
076            }
077        }
078    
079        private void addUpstreamProteinReferences(CCPTextAnnotation pronounAnnot, JCas jcas) {
080            /*
081             * Iterate through the semantic annotations and find all proteins that are upstream of the pronoun. This is not the most efficient
082             * way to do this, but for the sake of time...
083             */
084            List<CCPTextAnnotation> upstreamProteins = new ArrayList<CCPTextAnnotation>();
085            Iterator annotationIter = jcas.getJFSIndexRepository().getAnnotationIndex(CCPTextAnnotation.type).iterator();
086            while (annotationIter.hasNext()) {
087                CCPTextAnnotation sa = (CCPTextAnnotation) annotationIter.next();
088                if (sa.getClassMention().getMentionName().equals(ClassMentionTypes.PROTEIN) && sa.getEnd() < pronounAnnot.getBegin()) {
089                    /* Then this is a protein annotation upstream of the input TokenAnnotation (pronoun) so add it to the list */
090                    upstreamProteins.add(sa);
091                    if (DEBUG) {
092                        System.out.println("Found upstream protein: ");
093                        UIMA_Util.printCCPTextAnnotation(sa, System.out);
094                    }
095                }
096            }
097    
098            if (singularPronounList.contains(pronounAnnot.getCoveredText().toLowerCase())) {
099                if (tokenStartsAClause(pronounAnnot, jcas)) {
100                    /* return the first protein in the sentence ( = first protein in the list) */
101                    addProteinsFromListToJCas(ADD_FIRST_SINGULAR, upstreamProteins, pronounAnnot, jcas);
102                } else {
103                    addProteinsFromListToJCas(ADD_LAST_SINGULAR, upstreamProteins, pronounAnnot, jcas);
104                }
105            } else if (pluralPronounList.contains(pronounAnnot.getCoveredText().toLowerCase())) {
106                if (tokenStartsAClause(pronounAnnot, jcas)) {
107                    addProteinsFromListToJCas(ADD_FIRST_PLURAL, upstreamProteins, pronounAnnot, jcas);
108                } else {
109                    addProteinsFromListToJCas(ADD_LAST_PLURAL, upstreamProteins, pronounAnnot, jcas);
110                }
111            }
112    
113        }
114    
115        /*
116         * Returns new protein annotations
117         */
118        private void addProteinsFromListToJCas(int addType, List<CCPTextAnnotation> proteinList, CCPTextAnnotation pronounAnnot, JCas jcas) {
119            List<CCPTextAnnotation> proteinsToAdd = new ArrayList<CCPTextAnnotation>();
120    
121            if (proteinList.size() > 0) {
122                /* If there are known upstream protein annotations */
123                if (addType == ADD_FIRST_SINGULAR) {
124                    if (DEBUG) {
125                        System.out.println("Adding first singular...");
126                    }
127                    /* Add the first protein in the list if its POS is singular */
128                    CCPTextAnnotation referencedProtein = proteinList.get(0);
129                    List<CCPTextAnnotation> proteinTokens = getProteinTokens(referencedProtein, jcas);
130                    if (DEBUG) {
131                        System.out.println("Checking for plurarity in token: " + proteinTokens.get(proteinTokens.size()-1).getCoveredText());
132                    }
133                    /* Look at the far right token (if there are more than 1 token, and check to see that the POS is singular */
134    //                String pos = UIMA_Util.getFirstSlotValue((CCPNonComplexSlotMention) UIMA_Util.getSlotMentionByName(proteinTokens
135    //                        .get(proteinTokens.size() - 1), SlotMentionTypes.TOKEN_PARTOFSPEECH));
136    //                if (isPartOfSpeechSingular(pos)) {
137                        proteinsToAdd.add(referencedProtein);
138    //                }
139                } else if (addType == ADD_LAST_SINGULAR) {
140                    if (DEBUG) {
141                        System.out.println("Adding last singular...");
142                    }
143                    /* Add the last protein in the list if its POS is singular */
144                    CCPTextAnnotation referencedProtein = proteinList.get(proteinList.size() - 1);
145                    List<CCPTextAnnotation> proteinTokens = getProteinTokens(referencedProtein, jcas);
146                    
147                    if (DEBUG) {
148                        System.err.println("Size of protein tokens list: " + proteinTokens.size());
149                        System.err.println("REFERENCED PROTEIN: ");
150                        UIMA_Util.printCCPTextAnnotation(referencedProtein, System.err);
151                            System.out.println("Checking for plurarity in token: " + proteinTokens.get(proteinTokens.size()-1).getCoveredText());
152                    }
153                    /* Look at the far right token (if there are more than 1 token, and check to see that the POS is singular */
154    //                String pos = UIMA_Util.getFirstSlotValue((CCPNonComplexSlotMention) UIMA_Util.getSlotMentionByName(proteinTokens
155    //                        .get(proteinTokens.size() - 1), SlotMentionTypes.TOKEN_PARTOFSPEECH));
156    //                if (isPartOfSpeechSingular(pos)) {
157                        proteinsToAdd.add(referencedProtein);
158    //                }
159                } else if (addType == ADD_FIRST_PLURAL) {
160                    if (DEBUG) {
161                        System.out.println("Adding first plural...");
162                    }
163                    /* Add the first protein in the list if its POS is plural */
164                    CCPTextAnnotation referencedProtein = proteinList.get(0);
165                    List<CCPTextAnnotation> proteinTokens = getProteinTokens(referencedProtein, jcas);
166                    /* Look at the far right token (if there are more than 1 token, and check to see that the POS is singular */
167    //                if (DEBUG) {
168    //                    System.out.println("Checking for plurarity in token: " + proteinTokens.get(proteinTokens.size()-1).getCoveredText());
169    //                }
170    //                String pos = UIMA_Util.getFirstSlotValue((CCPNonComplexSlotMention) UIMA_Util.getSlotMentionByName(proteinTokens
171    //                        .get(proteinTokens.size() - 1), SlotMentionTypes.TOKEN_PARTOFSPEECH));
172    //                if (!isPartOfSpeechSingular(pos)) {
173    //                    proteinsToAdd.add(referencedProtein);
174    //                } else {
175                        /* Add the first two proteins in the list if they are separated by "and" */
176                        if (proteinList.size() > 1) {
177                            /* make sure there is more than one upstream protein */
178                            CCPTextAnnotation referencedProtein_2 = proteinList.get(1);
179                            List<CCPTextAnnotation> proteinTokens_2 = getProteinTokens(referencedProtein_2, jcas);
180                            /* Look at the far right token (if there are more than 1 token, and check to see that the POS is singular */
181    //                        if (DEBUG) {
182    //                            System.out.println("Checking for plurarity in token: " + proteinTokens.get(proteinTokens.size()-1).getCoveredText());
183    //                        }
184    //                        pos = UIMA_Util.getFirstSlotValue((CCPNonComplexSlotMention) UIMA_Util.getSlotMentionByName(proteinTokens_2
185    //                                .get(proteinTokens_2.size() - 1), SlotMentionTypes.TOKEN_PARTOFSPEECH));
186    //                        if (isPartOfSpeechSingular(pos)) {
187                                /* If the second protein is also singular, check to see if the two proteins are separated by "and" */
188                                if (annotationsAreSeparatedBy("and", referencedProtein_2, referencedProtein, jcas)) {
189                                    proteinsToAdd.add(referencedProtein);
190                                    proteinsToAdd.add(referencedProtein_2);
191                                } else {
192                                    proteinsToAdd.add(referencedProtein_2);
193                                }
194    //                        }
195                        } else {
196                            proteinsToAdd.add(referencedProtein);
197                    }
198                } else if (addType == ADD_LAST_PLURAL) {
199                    if (DEBUG) {
200                        System.out.println("Adding last plural...");
201                    }
202                    /* Add the last protein in the list if its POS is plural */
203                    CCPTextAnnotation referencedProtein = proteinList.get(proteinList.size() - 1);
204                    List<CCPTextAnnotation> proteinTokens = getProteinTokens(referencedProtein, jcas);
205                    /* Look at the far right token (if there are more than 1 token, and check to see that the POS is singular */
206    //                if (DEBUG) {
207    //                    System.out.println("Checking for plurarity in token: " + proteinTokens.get(proteinTokens.size()-1).getCoveredText());
208    //                }
209    //                String pos = UIMA_Util.getFirstSlotValue((CCPNonComplexSlotMention) UIMA_Util.getSlotMentionByName(proteinTokens
210    //                        .get(proteinTokens.size() - 1), SlotMentionTypes.TOKEN_PARTOFSPEECH));
211    //                if (!isPartOfSpeechSingular(pos)) {
212    //                    proteinsToAdd.add(referencedProtein);
213    //                } else {
214    //                    /* Add the last two proteins in the list if they are separated by "and" */
215                        if (proteinList.size() > 1) {
216                            /* make sure there is more than one upstream protein */
217                            CCPTextAnnotation referencedProtein_2 = proteinList.get(proteinList.size() - 2);
218                            List<CCPTextAnnotation> proteinTokens_2 = getProteinTokens(referencedProtein_2, jcas);
219                            /* Look at the far right token (if there are more than 1 token, and check to see that the POS is singular */
220                            if (DEBUG) {
221                                System.out.println("Checking for plurarity in token: " + proteinTokens.get(proteinTokens.size()-1).getCoveredText());
222                            }
223    //                        pos = UIMA_Util.getFirstSlotValue((CCPNonComplexSlotMention) UIMA_Util.getSlotMentionByName(proteinTokens_2
224    //                                .get(proteinTokens_2.size() - 1), SlotMentionTypes.TOKEN_PARTOFSPEECH));
225    //                        if (isPartOfSpeechSingular(pos)) {
226    //                            /* If the second protein is also singular, check to see if the two proteins are separated by "and" */
227                                if (annotationsAreSeparatedBy("and", referencedProtein, referencedProtein_2, jcas)) {
228                                    proteinsToAdd.add(referencedProtein);
229                                    proteinsToAdd.add(referencedProtein_2);
230                                } else {
231                                    proteinsToAdd.add(referencedProtein_2);
232                            }
233                        } else {
234                            proteinsToAdd.add(referencedProtein);
235                        }
236                    
237                }
238    
239                
240                /* Get document ID */
241                String docID = "";
242                Iterator docInfoIterator = jcas.getJFSIndexRepository().getAnnotationIndex(CCPDocumentInformation.type).iterator();
243                if (docInfoIterator.hasNext()) {
244                    CCPDocumentInformation ccpDocInfo = (CCPDocumentInformation) docInfoIterator.next();
245                    docID = ccpDocInfo.getDocumentID();
246                }
247                
248                for (CCPTextAnnotation referencedProtein : proteinsToAdd) {
249                    /* create a new protein annotation with the same span as the pronoun, and add it to the JCas */
250                    CCPTextAnnotation newProteinAnnotation = new CCPSemanticAnnotation(jcas);
251                    try {
252                    UIMA_Util.swapAnnotationInfo(referencedProtein, newProteinAnnotation);
253                    } catch (CASException ce) {
254                        ce.printStackTrace();
255                    }
256                    /* set the begin and end, and span list equal to that of the pronoun */
257                    newProteinAnnotation.setBegin(pronounAnnot.getBegin());
258                    newProteinAnnotation.setEnd(pronounAnnot.getEnd());
259                    newProteinAnnotation.setSpans(pronounAnnot.getSpans());
260                    //newProteinAnnotation.addToIndexes();
261                    proteinsToAddToCAS.add(newProteinAnnotation);
262                    if (DEBUG) {
263                        System.out.println("%%%%%%%%%%%%%%%%%  New protein annotation %%%%%%%%%%%%%%%%%%%%");
264                        UIMA_Util.printCCPTextAnnotation(newProteinAnnotation, System.out);
265                        
266                    }
267                    
268    //                printOutputString(newProteinAnnotation, docID);
269                }
270    
271            }
272        }
273    
274        private void printOutputString(CCPTextAnnotation ccpTA, String docID){
275        
276        CCPTextAnnotation antecedentAnnotation = (CCPTextAnnotation)ccpTA.getClassMention().getCcpTextAnnotations().get(0);
277        String antecedentText = antecedentAnnotation.getCoveredText();
278        String antecedentSpan = "[" + antecedentAnnotation.getBegin() + ".." + antecedentAnnotation.getEnd() + "]";
279        String anaphorText = ccpTA.getCoveredText();
280        String anaphorSpan = "[" + ccpTA.getBegin() + ".." + ccpTA.getEnd() + "]";
281        
282        String outStr = docID + "|" + antecedentText + antecedentSpan + "{antecedent}|" + anaphorText + anaphorSpan + "{anaphor}";
283        
284        System.out.println(outStr);
285        
286        }
287        
288        /*
289         * returns true if the two input annotations are separated only by the separationText
290         */
291        private boolean annotationsAreSeparatedBy(String separationText, CCPTextAnnotation upstreamAnnot, CCPTextAnnotation downstreamAnnot,
292                JCas jcas) {
293    
294            Span upstreamSpan = null;
295            Span downstreamSpan = null;
296    
297            try {
298                upstreamSpan = new Span(upstreamAnnot.getBegin(), upstreamAnnot.getEnd());
299                downstreamSpan = new Span(downstreamAnnot.getBegin(), downstreamAnnot.getEnd());
300            } catch (InvalidSpanException e) {
301                e.printStackTrace();
302            }
303    
304            Iterator<CCPTextAnnotation> annotationsBetweenSpansIter = UIMA_Util
305                    .getAnnotationsInBetweenSpans(upstreamSpan, downstreamSpan, jcas);
306            if (annotationsBetweenSpansIter == null) {
307                return false;
308            } else {
309            int annotCount = 0;
310            boolean isSeparationText = false;
311            while (annotationsBetweenSpansIter.hasNext()) {
312                CCPTextAnnotation ccpTA = (CCPTextAnnotation) annotationsBetweenSpansIter.next();
313    
314                if (ccpTA.getCoveredText().equalsIgnoreCase(separationText)) {
315                    isSeparationText = true;
316                }
317                annotCount++;
318            }
319    
320            /* If there is only one token between input annotations, and that token equals the separationText, then return true. */
321            if (annotCount == 1 && isSeparationText) {
322                return true;
323            } else {
324                return false;
325            }
326            }
327        }
328    
329        /*
330         * Not certain this is a good assumption, but if the POS ends in an "s", then we consider it plural, otherwise it is singular
331         */
332        private boolean isPartOfSpeechSingular(String posStr) {
333            if (posStr.toLowerCase().endsWith("s")) {
334                if (DEBUG) {
335                    System.out.println("Part of Speech: " + posStr + " is NOT singular");
336                }
337                return false;
338            } else {
339                if (DEBUG) {
340                    System.out.println("Part of Speech: " + posStr + " is singular");
341                }
342                return true;
343            }
344        }
345    
346        /*
347         * returns the token annotations for a given protein annotation
348         */
349        private List<CCPTextAnnotation> getProteinTokens(CCPTextAnnotation proteinAnnot, JCas jcas) {
350            Span proteinSpan = null;
351            try {
352                proteinSpan = new Span(proteinAnnot.getBegin(), proteinAnnot.getEnd());
353            } catch (InvalidSpanException e) {
354                e.printStackTrace();
355            }
356    
357            List<CCPTextAnnotation> proteinTokens = new ArrayList<CCPTextAnnotation>();
358            Iterator<CCPTextAnnotation> annotationsWithinSpanIter = UIMA_Util.getAnnotationsEncompassingSpan(proteinSpan, jcas);
359            while (annotationsWithinSpanIter.hasNext()) {
360                CCPTextAnnotation ccpTA = (CCPTextAnnotation) annotationsWithinSpanIter.next();
361                if (ccpTA.getClassMention().getMentionName().equals(ClassMentionTypes.TOKEN)) {
362                    proteinTokens.add(ccpTA);
363                }
364            }
365    
366            return proteinTokens;
367    
368        }
369    
370        /*
371         * Determines if the input TokenAnnotation is at the beginning of a Clause
372         */
373        private boolean tokenStartsAClause(CCPTextAnnotation tokenAnnot, JCas jcas) {
374            /* If starts with a capital letter */
375            String tokenText = tokenAnnot.getCoveredText(); 
376            if (Character.isUpperCase(tokenText.charAt(0))) {
377                return true;
378            } else {
379                return false;
380            }
381          
382            
383            
384    //        /* Get an iterator over all annotations that begin with the same starting index as the input TokenAnnotation */
385    //        Iterator<CCPTextAnnotation> annotationsWithSameStartIter = UIMA_Util.getAnnotationsWithSameStart(tokenAnnot.getBegin(), jcas);
386    //        while (annotationsWithSameStartIter.hasNext()) {
387    //            CCPTextAnnotation ccpTA = (CCPTextAnnotation) annotationsWithSameStartIter.next();
388    //
389    //            if (ccpTA.getClassMention().getMentionName().equals(ClassMentionTypes.CLAUSE)) {
390    //                /* If there is a CLAUSE that begins at the same index as the TokenAnnotation, then return true */
391    //               if (DEBUG) {
392    //                   System.out.println("Token: " + tokenAnnot.getCoveredText() + " starts a clause!");
393    //               }
394    //                return true;
395    //            }
396    //
397    //        }
398    //
399    //        if (DEBUG) {
400    //            System.out.println("Token: " + tokenAnnot.getCoveredText() + " does NOT start a clause!");
401    //        }
402    //        /* No Clause was found to start at the same place as the TokenAnnotation so return false */
403    //        return false;
404    
405        }
406    
407    }
```
